# Standardized mortality ratios for regionalized acute cardiovascular care

**DOI:** 10.1186/s12913-023-09883-w

**Published:** 2023-09-05

**Authors:** Sanne J. den Hartog, Bob Roozenbeek, Sjoukje van der Bij, Marzyeh Amini, Nikki van Leeuwen, Eric Boersma, Clemens M.F. Dirven, Diederik W.J. Dippel, Hester F. Lingsma

**Affiliations:** 1 Department of Neurology, Erasmus MC, University Medical Center, Rotterdam, The Netherlands; 2 Department of Radiology and Nuclear Medicine, Erasmus MC, University Medical Center, Rotterdam, The Netherlands; 3 Department of Public Health, Erasmus MC, University Medical Center, Rotterdam, The Netherlands; 4 Dutch Hospital Data, Utrecht, The Netherlands; 5 Department of Cardiology, Erasmus MC, University Medical Center Rotterdam, Rotterdam, The Netherlands; 6 Department of Neurosurgery, Erasmus MC, University Medical Center, Rotterdam, The Netherlands; 7 Erasmus MC, Department of Neurology, Department of Radiology and Nuclear Medicine, Department of Public Health, University Medical Center, Room Ee2240, 3000 CA Rotterdam, P.O. Box 2040, the Netherlands

**Keywords:** Quality of care, Cardiovascular care, Quality indicators

## Abstract

**Background:**

Standardized Mortality Ratios (SMRs) are case-mix adjusted mortality rates per hospital and are used to evaluate quality of care. However, acute care is increasingly organized on a regional level, with more severe patients admitted to specialized hospitals. We hypothesize that the current case-mix adjustment insufficiently captures differences in case-mix between non-specialized and specialized hospitals. We aim to improve the SMR by adding proxies of disease severity to the model and by calculating a regional SMR (RSMR) for acute cerebrovascular disease (CVD) and myocardial infarction (MI).

**Methods:**

We used data from the Dutch National Basic Registration of Hospital Care. We selected all admissions from 2016 to 2018. SMRs and RSMRs were calculated by dividing the observed in-hospital mortality by the expected in-hospital mortality. The expected in-hospital mortality was calculated using logistic regression with adjustment for age, sex, socioeconomic status, severity of main diagnosis, urgency of admission, Charlson comorbidity index, place of residence before admission, month/year of admission, and in-hospital mortality as outcome.

**Results:**

The IQR of hospital SMRs of CVD was 0.85–1.10, median 0.94, with higher SMRs for specialized hospitals (median 1.12, IQR 1.00-1.28, 71%-SMR > 1) than for non-specialized hospitals (median 0.92, IQR 0.82–1.07, 32%-SMR > 1). The IQR of RSMRs was 0.92–1.09, median 1.00. The IQR of hospital SMRs of MI was 0.76–1.14, median 0.98, with higher SMRs for specialized hospitals (median 1.00, IQR 0.89–1.25, 50%-SMR > 1 versus median 0.94, IQR 0.74–1.11, 44%-SMR > 1). The IQR of RSMRs was 0.90–1.08, median 1.00. Adjustment for proxies of disease severity mostly led to lower SMRs of specialized hospitals.

**Conclusion:**

SMRs of acute regionally organized diseases do not only measure differences in quality of care between hospitals, but merely measure differences in case-mix between hospitals. Although the addition of proxies of disease severity improves the model to calculate SMRs, real disease severity scores would be preferred. However, such scores are not available in administrative data. As a consequence, the usefulness of the current SMR as quality indicator is very limited. RSMRs are potentially more useful, since they fit regional organization and might be a more valid representation of quality of care.

**Supplementary Information:**

The online version contains supplementary material available at 10.1186/s12913-023-09883-w.

## Introduction

Standardized Mortality Ratios (SMRs) are ratios of observed and expected numbers of deaths per hospital and are frequently used to evaluate quality of hospital care. These SMRs can be used as an alert function and trigger for quality improvement, because high SMRs may reflect general problems concerning quality of care in a hospital [1]. In the Netherlands the hospitals are required to make SMRs public. The SMRs are also used in several other countries like USA, Canada, Sweden, Wales, Australia, France, and Japan [2].

Previously, the validity of the SMRs as an indicator of quality of care was questioned [3–10]. To be able to compare hospital performance, it is needed to adjust for differences in patient characteristics (case-mix) between hospitals. Specialized hospitals often treat patients with a more severe disease and so a higher baseline risk of mortality [3]. Insufficient case-mix adjustment will lead to higher SMRs of specialized hospitals, while they might provide good quality of care [3, 10]. In addition, specialized hospitals receive patients from non-specialized hospitals for specific treatments/interventions. These transferred patients often have more advanced diseases and a higher chance of mortality [11]. The specialized hospital that admit the transferred patients will be negatively affected when a patient dies during the hospital stay, whereas the referring hospital is given a positive outcome (the patient left that hospital still alive) [3, 10]. Such ‘referral bias’ can make the apparent differences in SMRs between specialized and non-specialized hospitals even larger. We hypothesize that the current case-mix adjustment insufficiently captures differences in case-mix between specialized and non-specialized hospitals. Therefore, a regional measured SMR could be a more valid and useful measure of quality of care. Within the regions there is usually a collaboration of specialized and non-specialized hospitals and agreement on where a patient with a specific disease will be treated. These hospitals have a shared responsibility for the acute treatment of a patient. We aim to compare hospital SMRs, for specialized and non-specialized hospitals for two acute diseases for which care is regionally organized: acute cerebrovascular disease and acute myocardial infarction. To better capture the differences in SMRs between specialized and non-specialized hospitals in case-mix, we investigated the influence of additional adjustment for proxies of disease severity. To take into account regionalization in the evaluation of hospital care, we calculated regional SMRs (RSMRs).

## Methods

### Study design and population

We conducted an observational retrospective cohort study using data from the Dutch National Basic Registration of Hospital Care (LBZ). This database provides data from all general and university hospitals in the Netherlands and contains all hospital admissions. Patients were allocated to diagnostic groups, which are clusters of ICD codes registered in the LBZ [12]. Here the ICD code of the main diagnosis of the admission is used, i.e. the main reason for the hospital stays, which is determined at discharge. In total, 157 diagnostic groups are distinguished. We used the diagnostic groups acute cerebrovascular disease and acute myocardial infarction. We selected all admissions with a discharge date from 1 January  2016 to 31 December 2018. Patients admitted to different hospitals in the same period of time (e.g. transferred patients), were included in every hospital they were admitted. Hospitals were classified as specialized or non-specialized based on the provision of specialized treatments. For cerebrovascular disease, specialized hospitals provide endovascular thrombectomy (EVT) and for myocardial infarction specialized hospitals provide cardiac surgery and endovascular interventions. Acute cerebrovascular disease contains a heterogeneous group of diagnoses, therefore we made a breakdown of the specialized hospitals into endovascular capable centers and comprehensive stroke centers. The patients admitted in two oncology hospitals were excluded from analysis, because in these hospitals there were a very low number of admissions with acute cerebrovascular disease, or acute myocardial infarction. Hospitals with no admissions, only an outpatient clinic, were excluded from analysis and patients not living in the Netherlands were also excluded.

### Hospital SMR

We used the same approach as Statistics Netherlands uses to calculate the SMR for each diagnostic group [12]. For the selected diagnostic groups, a prediction model was used to calculate the expected probability of mortality of an admission, with data from all hospitals, adjusted for case-mix. Case-mix adjustment was based on logistic regression models with in-hospital mortality as the dependent variable and age, sex, socioeconomic status (based on the postal code of the patients’ residence), severity of main diagnosis (risk of mortality/probability of death based on historical data), urgency of admission (elective or acute), Charlson comorbidity index, place of residence before admission, and month/year of admission as predictor variables. These models produced an expected mortality probability for each admission. Adding up the probabilities of mortality per hospital gave the total expected number of deaths for that hospital. With this expected mortality number, we calculated the SMR.

$${\text{SMR}}\,dh\,{\text{ = }}\,\frac{{{\text{Observed}}\,{\text{mortality}}\,dh}}{{{\text{Expected}}\,{\text{mortality}}\,dh}}$$

The numerator was the observed number of deaths with main diagnosis *d* in hospital *h*. The.

denominator was the expected number of deaths for this type of diagnosis. An SMR of 1 means that the observed in-hospital mortality is the same as the expected in-hospital mortality. An SMR of < 1 is better than expected and > 1 is worse than expected.

Statistics Netherlands reports a case-mix model performance of cerebrovascular disease C-statistic 0.81, and myocardial infarction C-statistic 0.85.

### Regional SMR

Regions were determined by the Dutch regional network of acute care (n = 11). Each region contains at least one specialized hospital. The RSMR was calculated with the same regression model as the SMR calculated on a hospital level. This regression model estimates the probability of mortality per patient, which can be summed up per region to get the expected number of deaths. The RSMR can be calculated from this expected number and the observed mortality.

### Severity adjustment

We investigated differences in SMRs between specialized and non-specialized hospitals. We hypothesized that these differences are due to differences in case-mix, especially in the severity of the main diagnosis, between hospitals, as previously has been reported [3, 10]. Real severity scores were not available in administrative data. To adjust for severity of the main diagnosis we used specific treatments or interventions performed during admission, registered in the administrative data, as a proxy of disease severity. For every diagnostic group we added these proxies, separately, to the current case-mix adjustment model to investigate their influence on the SMR. For all patients we investigated the influence of inter-hospital transfer and intensive care unit (ICU) admission on the SMR. Inter-hospital transfer was defined as admission or presentation in a general hospital followed by presentation or admission to a specialized hospital within one day. The inter-hospital transfer was only counted for the receiving specialized hospital, since the SMR of the receiving hospital will be negatively affected when the patient dies during the hospital stay. ICU admission was defined as ICU admission in the first two days after admission. For patients with acute cerebrovascular disease we additionally investigated the influence of adding EVT to the case-mix adjustment model. For patients with acute myocardial infarction we investigated the influence of a clinical percutaneous coronary intervention (PCI) and coronary artery bypass grafting (CABG).

### Statistical analysis and missing data

We used descriptive statistics to compare the case-mix variables, proxies of diseases severity, and outcomes between specialized and non-specialized hospitals. We reported the interquartile ranges (IQR) and median of SMRs per disease separately for specialized and non-specialized hospitals. In addition, we reported the percentage of SMRs above 1 (higher mortality than expected) for specialized and non-specialized hospitals.

We investigated the influence of additional adjustment for proxies of disease severity on the SMR. In these proxies there were some missing values. These were imputed using single imputation with R based on relevant covariates and outcomes. We performed logistic regression analyses to investigate the association of proxies of disease severity with in-hospital mortality and presented (adjusted) odds ratios (ORs) with 95% confidence intervals (CI). We compared the current case-mix adjustment model with a model with additional adjustment with proxies of disease severity. Performance of the different models was expressed as the area under the receiver operating characteristic (ROC) curve (AUC). To take into account uncertainty due to low numbers of deaths per hospital or region, we performed a sensitivity analysis in which we used a mixed effects logistic regression model (GLMM) with a random intercept for hospital to estimate the SMR and a random intercept for region to estimate the RSMR. We compare these two models to examine whether and how the SMR changes by changing the model. All statistical analyses were performed with R statistical software (version 3.6.1).

## Results

### Acute cerebrovascular disease

We included 110.155 admissions with acute cerebrovascular disease (Table 1). The most common diagnoses of these admissions were acute cerebral infarction, 86.343 (78%), intracerebral haemorrhage, 13.401 (12%), and subarachnoid haemorrhage 4.698 (4%) (Supplement table SI). There were no clear differences in the percentages of the case-mix variables between specialized and non-specialized hospitals (Table 1). Patients in specialized hospitals had a more severe disease compared with non-specialized hospitals, as indicated by the proxies of disease severity. In specialized hospitals, 8% of the patients were transferred from a general hospital. There were more ICU admissions in specialized hospitals (13%) compared with non-specialized hospitals (4%). EVT was only performed in specialized hospitals (11%). Overall, the in-hospital mortality was 9%.

**Table 1:** Characteristics of all admissions with acute cerebrovascular disease and acute myocardial infarction between 2016–2018

	Cerebrovasular disease		Myocardial infarction	
	Specialized hospital	Non specialized hospital	Specialized hospital	Non specialized hospital
Number of Admissions	39517	70638	34085	67144
Number of hospitals	17	62	16	63
**Case-mix**				
Age, years	72 (61–81)	75 (65–83)	67 (57–76)	68 (58–78)
Men	53%	52%	69%	66%
Severity* 0-0.01 0.01–0.02 0.02–0.05 0.05–0.1 0.1–0.2 0.2–0.3 0.3–0.4 0.4-1 Other	NA NA 6% 71% 5% 11% 7% 1% NA	NA NA 4% 82% 1% 8% 5% 0.3% NA	NA 47% 28% 24% NA NA NA NA NA	NA 60% 21% 19% NA NA NA NA NA
Urgency Elective Acute	6% 94%	8% 92%	8% 92%	11% 89%
Social economic status Highest Above average Average Below average Lowest Unknown†	17% 17% 22% 20% 23% 0.4%	15% 20% 20% 22% 23% 0.5%	18% 19% 20% 20% 23% 0.6%	15% 19% 21% 23% 22% 0.3%
Source Home Nursing home Hospital	89% 1% 10%	91% 1% 8%	79% 1% 20%	81% 2% 17%
Charlson comorbidity	55%	53%	44%	46%
**Treatments / Interventions**				
ICU admission first 2 days after admission	13%‡	4%	16%§	2%
Inter-hospital transfer	8% ||	0%	13%#	0%
EVT	11% **	0%	0%	0%
PCI	0%	0%	69%	29%
CABG	0%	0%	11% ††	0%
**Outcomes**				
Discharge destination Home Nursing home/hospice Rehabilitation Other institution Hospital Dead	48% 15% 8% 3% 15% 11%	56% 15% 14% 4% 4% 8%	57% 1% 0.4% 1% 37% 3%	75% 2% 1% 1% 19% 3%
In-hospital mortality	11%	8%	3%	3%

Categorical variables are presented as percentage, continuous variables are presented as median (IQR). NA, not applicable, ICU, intensive care unit, EVT, endovascular thrombectomy, PCI, percutaneous coronary intervention, CABG, coronary artery bypass grafting

*To classify the severity of the sub-diagnosis, we used the standard method suggested by Van den Bosch et al. (2011)[13], who suggested categorizing the ICD codes into mortality rate categories. Boundaries for the mortality rate intervals: 0, 0.01, 0.02, 0.05, 0.1, 0.2, 0.3, 0.4 and 1. (“0” means 0% mortality; “1” means 100% mortality). This is based on Dutch hospital mortality rates. The percentages indicate the probability of death with a given diagnosis derived from previous data

†missing is recoded as unknown

Number of missing values: ‡ 2489, § 3196, ||2684, #3879, **3220, ††4951

**Fig. 1:**
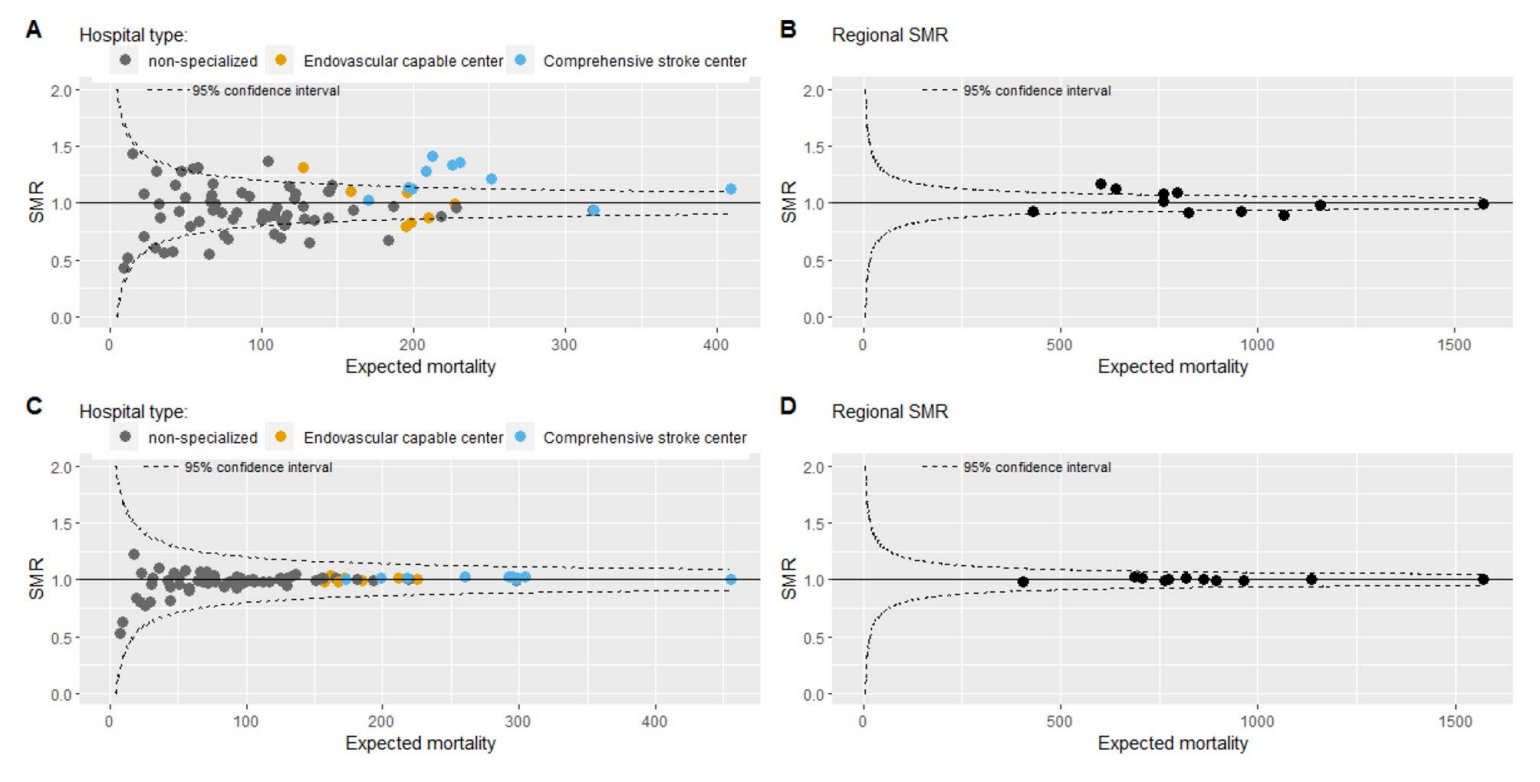
Standardized mortality ratio (SMR) of acute cerebrovascular disease. **(A)** calculated on a hospital level with a fixed effects model **(B)** calculated per region with a fixed effects model **(C)** calculated on a hospital level with a mixed effects model **(D)** calculated per region with a mixed effects model

The IQR of the SMR acute cerebrovascular disease per hospital was 0.85 to 1.10, median 0.94, (Fig. 1A), with higher SMRs for specialized hospitals (median 1.12, IQR 1.00-1.28, 71% SMR > 1) than for non-specialized hospitals (median 0.92, IQR 0.82–1.07, 32% SMR > 1). The IQR of the SMR per region was 0.92 to 1.09, median 1.00 (Fig. 1B).

Patients undergoing ICU admission, EVT, or inter-hospital transfer were more likely to die (aOR: 4.14 [95% CI: 3.86–4.43], aOR: 2.31 [95% CI: 2.06–2.55], aOR: 1.12 [95% CI: 0.98–1.27]) (Table 2). The addition of ICU admission or EVT to the model decreased the SMRs of specialized hospitals and increased the SMRs of non-specialized hospitals (Table 3). Additional adjustment with all proxies of disease severity improved the model (C-statistic: 0.83 (95%CI 0.827–0.835) versus C-statistic: 0.81 (95%CI 0.806–0.815)) compared with the current case-mix adjustment model. As expected the IQR of the hospital SMR and RSMR were smaller in the sensitivity analysis with the mixed effect models compared with the main analysis (Fig. 1C and D).

**Table 2:** Association between proxies of disease severity and in-hospital mortality in acute cerebrovascular disease and acute myocardial infarction

	Cerebrovascular disease		Myocardial infarction	
	Univariable model OR (95%CI)	Multivariable model aOR (95%CI)	Univariable model OR (95%CI)	Multivariable model aOR (95%CI)
ICU admission	4.39 (4.16–4.64)	4.14 (3.86–4.43)	6.11 (5.61–6.66)	7.35 (6.65–8.13)
Transfer	1.26 (1.13–1.41)	1.12 (0.98–1.27)	1.00 (0.83–1.19)	2.12 (1.73–2.61)
EVT	1.40 (1.27–1.54)	2.31 (2.06–2.55)		
PCI			0.57 (0.53–0.62)	0.68 (0.62–0.75)
CABG			1.12 (0.91–1.36)	1.87 (1.51–2.32)

The multivariable model contains the current case-mix variables: age, sex, socioeconomic status, severity of main diagnosis, urgency of admission, Charlson comorbidity index, place of residence before admission, and month/year of admission.

OR, odds ratio, aOR, adjusted odds ratio, CI, confidence interval, ICU, intensive care unit, EVT, endovascular treatment, PCI, percutaneous coronary intervention, CABG, coronary artery bypass grafting.

### Acute myocardial infarction

We included 101.229 admissions with acute myocardial infarction (Table 1) and the most frequent used ICD code was of acute subendocardial myocardial infarction (56%) (Table SII of the Data Supplement). There were differences in disease severity between specialized and non-specialized hospitals; in specialized hospitals, 13% of the patients were transferred from a general hospital. There were more ICU admissions in specialized hospitals (16%) compared with non-specialized hospitals (2%). PCI treatment was performed in 69% of the admissions in specialized hospitals compared to 29% of the admissions in non-specialized hospitals. CABG was only performed in specialized hospitals, in 11% of the admissions. Overall, the in-hospital mortality was 3%.

**Fig. 2:**
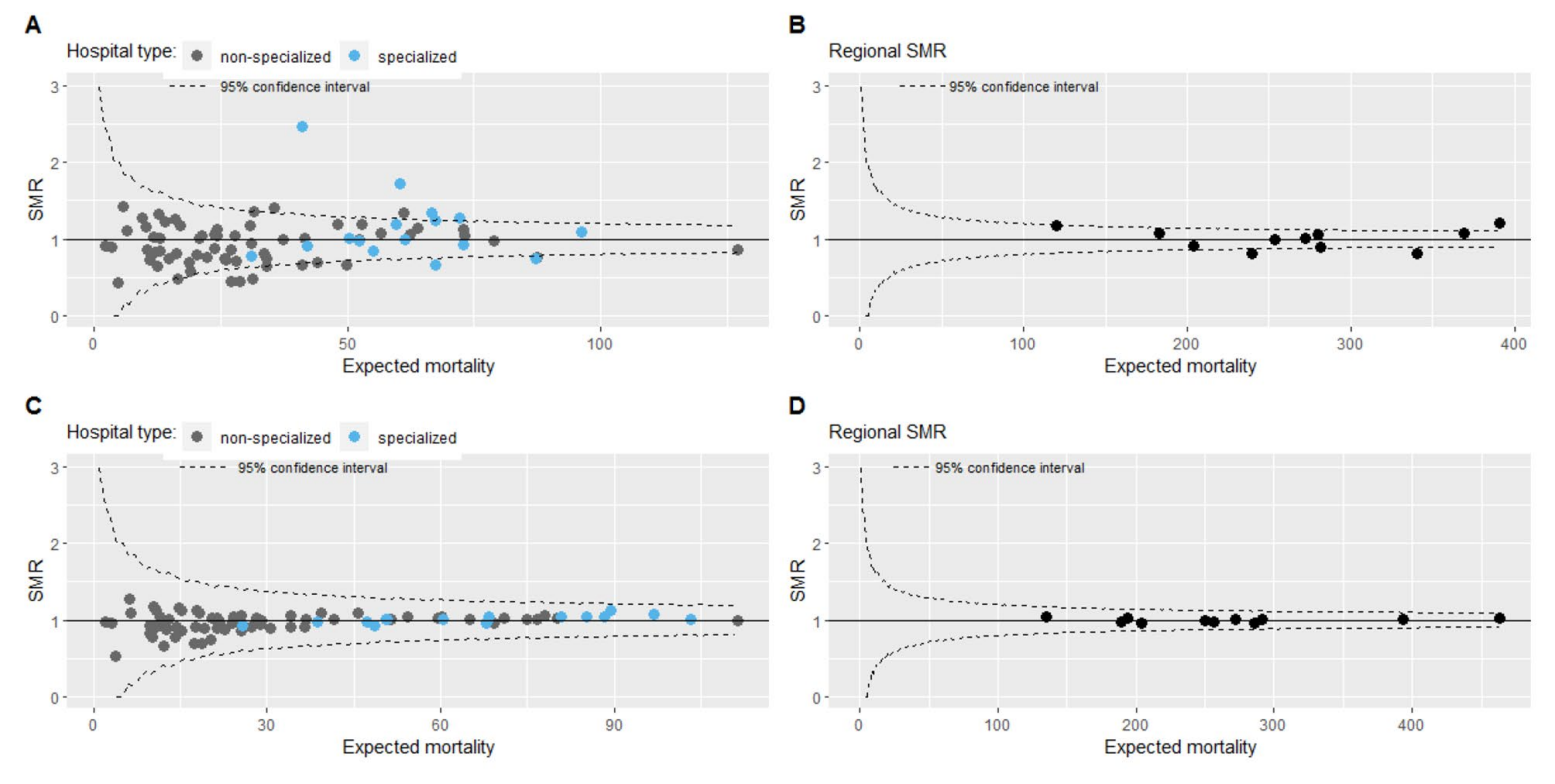
Standardized mortality ratio (SMR) of acute myocardial infarction. **(A)** calculated on a hospital level with a fixed effects model **(B)** calculated per region with a a fixed effects model **(C)** calculated on a hospital level with a mixed effects model **(D)** calculated per region with a mixed effects model

The IQR of the SMR acute myocardial infarction per hospital was 0.76–1.14, median 0.98 (Fig. 2A), with higher SMRs for specialized hospitals (median 1.00, IQR 0.89–1.25, 50% SMR > 1) than for non-specialized hospitals (median 0.94, IQR 0.74–1.11, 44% SMR > 1). The IQR of the SMRs per region was 0.90 to 1.08, median 1.00 (Fig. 2B).

Patients undergoing ICU admission, CABG or inter-hospital transfer were more likely to die (aOR: 7.35 [95% CI: 6.65–8.13], aOR: 1.87 [95% CI: 1.51–2.32], (aOR: 2.12 [95% CI: 1.73–2.61]) (Table 2). The addition of ICU admission, CABG, or inter-hospital transfer to the model decreased the SMRs of specialized hospitals, PCI did not (Table 3). Additional adjustment for all proxies of disease severity improved the model (C-statistic: 0.88 (95%CI 0.879–0.891) versus C-statistic: 0.85 (95%CI 0.843–0.856)) compared to the current case-mix adjustment model. Again, the IQRs of the hospital SMR and RSMR were smaller in the sensitivity analysis with the mixed effect models compared with the main analysis (Fig. 2C and D).

**Table 3:** The influence of adding proxies of disease severity to the case-mix adjustment model on the SMR

	Cerebrovascular disease Hospital SMR IQR			Myocardial infarction Hospital SMR IQR		
	All	Specialized	Non Specialized	All	Specialized	Non Specialized
Current case-mix model	0.85–1.10	1.00-1.28	0.82–1.07	0.76–1.14	0.89–1.25	0.74–1.11
ICU-admission	0.88–1.14	0.88–1.17	0.84–1.13	0.83–1.20	0.77–1.14	0.84–1.22
Transfer	0.85–1.10	0.99–1.28	0.82–1.07	0.76–1.15	0.83–1.21	0.76–1.13
EVT	0.86–1.11	0.95–1.21	0.84–1.09			
PCI				0.74–1.10	0.98–1.35	0.68–1.07
CABG				0.76–1.15	0.86–1.23	0.75–1.12
All above variables	0.87–1.14	0.88–1.15	0.85–1.14	0.76–1.19	0.92–1.25	0.73–1.17

IQR, interquartile range, SMR, standardized mortality ratio, ICU, intensive care unit, EVT, endovascular treatment, PCI, percutaneous coronary intervention, CABG, coronary artery bypass grafting

## Discussion

In this study we showed that between hospital differences in SMRs at least partly represent differences in case-mix between specialized and non-specialized hospitals instead of quality of care.

We showed large differences in SMRs between specialized and non-specialized hospitals in cerebrovascular disease and myocardial infarction. Most of the specialized hospitals had SMRs above 1. This could mean that the specialized hospitals provide poorer quality of care. However, we argue that it is more likely that these differences are due to differences in case-mix between the specialized and non-specialized hospitals. We tried to improve the case-mix adjustment model with additional adjustment for proxies of disease severity, because a real severity score is not available in administrative data. We do however advocate to improve case-mix adjustment with more specific disease scores instead of with procedures or proxies. Despite the strong association between these proxies and in-hospital mortality the change in SMRs was limited by adding these proxies to the model. This may mean that there are other differences between specialized and non-specialized hospitals not captured in our additional adjustment, or because of the fact that the number of patients to whom the proxies apply is low. Previous studies about the influence of transfer on the SMR showed that the adjustment for transfer barely changed the SMR [10]. The influence of cardiac procedures (PCI, CABG) on the SMR was also relatively small [14, 15]. In our study the impact of additional adjustment for these cardiac procedures on the SMR was relatively small. The proxies of disease severity could be influenced by practice variation and policy variation between hospitals. However, patients with cerebrovascular disease and myocardial infarction are treated conform strict guidelines.

Ideally, deaths are attributed to the hospital that is responsible to the undesirable outcome. In the US CMS (Centers for Medicare and Medicaid Services) reports the death of a transferred patient is only attributed to the referring hospital. This reduces referral bias but a death could also be the result of the quality in the receiving center. The cascade leading to death might have started in the referring hospital, during transport, or only in the receiving specialized hospital. In practice the actual scenario and thus no perfect solution exists. Therefor we attribute deaths in patients that were transferred to the receiving hospital and add ‘transfer’ to the model that produces the number of expected deaths.

The reason for transfer is also unknown. In general, more severe patients will be transferred, but patients at very high risk may not be transferred as they are unlikely to benefit from an intervention. In previous studies, the SMR barely changed after adjustment for inter-hospital transfer [10]. Apparently, transferred patients had similar mortality risk (profile) as patients who were directly admitted to the specialized hospital. One might speculate that the decision to transport patients is dominated by the expected benefits of further (invasive) treatment rather than the estimated mortality risk. The modest influence of cardiac procedures (PCI, CABG) on the SMR, and the low odds of death in patients undergoing PCI can also be explained in this way. Patient undergoing PCI are less severely diseased as patients undergoing CABG. PCI is probably not a good proxy of disease severity. A clinical severity score of the main diagnosis would be preferred to include in the adjustment models. For acute cerebrovascular disease the score on the National Institutes of Health Stroke Scale (NIHSS) is frequently used in clinical registries to indicate the severity of neurologic deficit [16]. For acute myocardial infarction the HEART score could be used. However, these scores are not available in administrative data that are used to calculate SMRs. Further research is needed to investigate the performance of the SMR model when adjustment with disease-specific severity scores were added to the model and whether this reduces the differences in SMRs between specialized and non-specialized hospitals.

A novel approach in our study was to calculate the SMR on a regional level. We showed that the ranges of the regional SMRs are much smaller than ranges of the hospital SMRs. When the SMR is measured on a regional level instead of a hospital level, differences in case-mix between specialized and non-specialized hospitals are less important, since there is a more equal distribution of high-risk patients over the regions, as compared to the distribution over hospitals. In addition, the smaller range can be explained by the statistical uncertainty which is due to a higher number of patients in a region compared to a hospital. The SMR in the Netherlands is currently estimated with fixed effect models. In such a model the estimates are based solely on the observed outcome in each hospital, which could lead to extreme estimates by chance [17]. A mixed effects model shrinks the hospital estimates to the mean, especially in case of small sample sizes, and results in more conservative estimates of ‘performance’ which we consider preferable in case of publicly reported performance estimates such as the SMRs. When we calculated the SMR with a mixed effects model and compared the SMR measured on a hospital level to the RSMR the differences are still present but smaller than in the main analysis.

Given the limitations, of the SMR measured on a hospital level, the RSMR could be a more useful quality indicator. Regional SMRs are more in line with the clinical practice of regionalized care and underlines the shared responsibility of multiple hospitals for the treatment of a patient. We described two acute diseases that are regionally organized and in which the regions are clearly defined with little interregional transfer. This makes it possible to compare regional care. Each region is allowed to decide how their care is arranged. Which implies that there are differences between regions in the care of a patients. We showed that there is only a small variation in SMRs between regions in the Netherlands, however the funnel plot showed that some RSMRs are outside the confidence intervals, so there is potential for improvement. Further research is needed to investigate the underlying causes of the variation between regions.

### Strengths and limitations

A strength of the study is the large number of patients we analysed. We used the same data as on which the current SMR is calculated and improved the model using available data, to ensure a direct potential to implement these adaptations.

A limitation of the study is the content of the diagnostic groups. Patients were divided into diagnostic groups, which are clusters of ICD codes. Acute cerebrovascular disease comprises: acute ischemic stroke, intracerebral haemorrhage, subarachnoid haemorrhage, acute nontraumatic subdural haemorrhage, and occlusion and stenosis of cerebral arteries not resulting in cerebral infarction. The probability of mortality differs between these diagnoses. For example, occlusion and stenosis of cerebral arteries not resulting in cerebral infarction (n = 364, 0.3%) do not lead directly to mortality and do not need acute care in contrast to a subarachnoid haemorrhage with a high probability of mortality. It could be questioned if all diagnoses are in the right diagnostic group. We did not change the content of the diagnostic groups, because otherwise it would be difficult to compare the regional SMR model to the current SMR adjustment model. Moreover, the number of patients with a non-acute diagnosis was very small. Length of stay could influence the inhospital mortality and also discharge bias could play a role, however we did not explicitly study this.

## Conclusion

SMRs in acute regionally organized diseases vary substantially and at least partly represent differences in case-mix between specialized and non-specialized hospitals instead of quality of care. Although the addition of proxies of disease severity improves the model to calculate SMRs, real disease severity scores would be preferred, However, such scores are not yet available in administrative data. As a consequence, the usefulness of the current SMR measured on a hospital level as quality indicator is very limited. RSMRs could be preferable over hospital SMRs, since they fit regional organization and might be a more valid representation of quality of care.

### Electronic supplementary material

Below is the link to the electronic supplementary material.

Supplementary Material 1

## Data Availability

The data that support the findings of this study are available from DHD but restrictions apply to the availability of these data, which were used under license for the current study, and so are not publicly available. Data are however available from the authors upon reasonable request and with permission of DHD.

## Abbreviations

CABG: coronary artery bypass grafting
CI: confidence interval
CVD: Cerebrovascular disease
DHD: Dutch Hospital Data
EVT: endovascular thrombectomy
ICU: intensive care unit
IQR: interquartile range
LBZ: Dutch National Basic Registration of Hospital Care
MI: myocardial infarction
OR: odds ratio
PCI: percutaneous coronary intervention
RSMR: Regional Standardized Mortality Ratio
SMR: Standardized Mortality Ratio
