# Supplementary material for: Standardized mortality ratios for regionalized acute cardiovascular care
Source: BMC Health Serv Res. 2023 Sep 5;23:951. doi: 10.1186/s12913-023-09883-w (PMC10481617; doi:10.1186/s12913-023-09883-w)
Supplement: Supplementary file 1 — Supplementary Material 1 [file 12913_2023_9883_MOESM1_ESM.pdf]

SUPPLEMENTAL MATERIAL

Standardized mortality ratios for regionalized acute cardiovascular care.

Sanne J. den Hartog, Bob Roozenbeek, Sjoukje van der Bij, Marzyeh Amini, Nikki van Leeuwen, Eric Boersma, Clemens M.F. Dirven, Diederik W.J. Dippel, Hester F. Lingsma

Table SI. Diagnosis group acute Cerebrovascular disease

ICD-10 code	Diagnoses	N (%)
I63.0-I63.6, I63.8-I64.0	Acute cerebral infarction	86343 (78%)
I61.0-I61.6, I61.8, I61.9	Intracerebral hemorrhage	13401 (12%)
I60.0-I60.9	Subarachnoid hemorrhage	4698 (4%)
I62.0, I62.1, I62.9	Subdural haemorrhage (acute)(nontraumatic)	5349 (5%)
I66.0-I66.4, I64.8, I64.9	Occlusion and stenosis of cerebral arteries, not resulting in cerebral infarction	364 (0.3%)

Table SII. Diagnosis group acute Myocardial infarction

ICD-10 code	Diagnoses	N (%)
I21	**Acute myocardial infarction**	
I21.0	Transmural infarction (acute) anteroapical	14445 (14%)
I21.1	Transmural infarction (acute) inferolateral	19073 (19%)
I21.2	Transmural infarction (acuut) basolateral	2511 (2%)
I21.3	Transmural myocardial infarct, not specified	4657 (5%)
I21.4	Acute subendocardial myocardial infarction	56259 (56%)
I21.9	Myocardial infarction (acute), not specified	3798 (4%)
I22	**Recurrent myocardial infarction**	
I22.0	Recurrent infarction (acute) anteroapical	62 (0.06%)
I22.1	Recurrent infarction (acute) inferolateral	85 (0.08%)
I22.8	Recurrent infarction (acute) basolateral	122 (0.1%)
I22.9	Recurrent myocardial infarction, not specified	217 (0.2%)
